# DNA Polymerases β and λ Mediate Overlapping and Independent Roles in Base Excision Repair in Mouse Embryonic Fibroblasts

**DOI:** 10.1371/journal.pone.0012229

**Published:** 2010-08-18

**Authors:** Elena K. Braithwaite, Padmini S. Kedar, Deborah J. Stumpo, Barbara Bertocci, Jonathan H. Freedman, Leona D. Samson, Samuel H. Wilson

**Affiliations:** 1 Laboratory of Structural Biology, National Institute of Environmental Health Sciences, National Institutes of Health, Research Triangle Park, North Carolina, United States of America; 2 Laboratory of Toxicology and Pharmacology, National Institute of Environmental Health Sciences, National Institutes of Health, Research Triangle Park, North Carolina, United States of America; 3 Laboratory of Signal Transduction, National Institute of Environmental Health Sciences, National Institutes of Health, Research Triangle Park, North Carolina, United States of America; 4 Institut National de la Santé et de la Recherche Médicale, Unité 783, Faculté de Médecine Paris Descartes, Paris, France; 5 Biological Engineering Department, Massachusetts Institute of Technology, Cambridge, Massachusetts, United States of America; University of Medicine and Dentistry of New Jersey, United States of America

## Abstract

Base excision repair (BER) is a DNA repair pathway designed to correct small base lesions in genomic DNA. While DNA polymerase beta (pol β) is known to be the main polymerase in the BER pathway, various studies have implicated other DNA polymerases in back-up roles. One such polymerase, DNA polymerase lambda (pol λ), was shown to be important in BER of oxidative DNA damage. To further explore roles of the X-family DNA polymerases λ and β in BER, we prepared a mouse embryonic fibroblast cell line with deletions in the genes for both pol β and pol λ. Neutral red viability assays demonstrated that pol λ and pol β double null cells were hypersensitive to alkylating and oxidizing DNA damaging agents. *In vitro* BER assays revealed a modest contribution of pol λ to single-nucleotide BER of base lesions. Additionally, using co-immunoprecipitation experiments with purified enzymes and whole cell extracts, we found that both pol λ and pol β interact with the upstream DNA glycosylases for repair of alkylated and oxidized DNA bases. Such interactions could be important in coordinating roles of these polymerases during BER.

## Introduction

Cells are constantly exposed to environmental and endogenous stressors such as reactive oxygen and nitrogen species, alkylating molecules and other reactive metabolites that are capable of damaging DNA. During replication and repair, DNA lesions induced by genotoxic compounds can encode for alternate nucleotides, potentially leading to permanent modifications in the genetic material. If these changes alter the function of key proteins required to regulate cell cycle progression or cellular defense mechanisms, adverse consequences for the cell may result.

Fortunately, cells maintain clever mechanisms by which they protect themselves from the detrimental effects of genotoxic compounds. Base excision repair (BER) is considered the predominant defense system for eliminating DNA lesions generated by alkylating agents, reactive oxygen species and spontaneous base loss or strand breakage in mammalian cells. Although there are at least two BER sub-pathways, the simplest BER sub-pathway results in replacement of the modified nucleotide only and is termed ‘single-nucleotide’ BER (SN BER). During SN BER, repair may be initiated by a DNA glycosylase, a specialized enzyme that recognizes specific types of DNA damage and removes the damaged base from the DNA phosphodiester backbone. The resulting apurinic/apyrimidinic (AP) site is cleaved by AP endonuclease 1 (APE1), producing a single-strand DNA break. DNA polymerase-mediated DNA synthesis and 5′-deoxyribose phosphate group (dRP) removal leads to a substrate for DNA ligase that completes SN BER. Since several mutagenic and cytotoxic intermediates are formed during BER, it is important that the process proceed efficiently to completion once the pathway is initiated [1], [2], [3].

While DNA polymerase beta (pol β) is thought to be the main polymerase involved in BER of lesions generated by monofunctional alkylating agents and reactive oxygen species in higher organisms, it is clear that other polymerases participate in this process to maintain genomic stability. DNA polymerase lambda (pol λ) is one such alternate polymerase that participates in the BER process. While pol λ, unlike pol β, is not required for survival in mice, it appears that pol λ can partially substitute for pol β during BER processing of DNA lesions, especially those from oxidative stress. Evidence supporting this statement came from biochemical experiments and genetic experiments in chicken DT40 cells, as well as from pol λ siRNA knockdown in mouse fibroblasts [4], [5]. These experiments, however, failed to evaluate the effect of a complete knockout of the pol λ gene in a mouse cell line with pol β null background.

Recently, interest in pol λ has been sparked by the observation that its error-free lesion bypass activity for the oxidized base 8-oxoguanine (8-oxodG) was strongly increased by the auxiliary factors PCNA and RPA [6], [7]. A similar alteration in the activity of pol β was not found. Although pol λ and pol β appear to have overlapping roles in BER, at least to some extent, it is likely that mechanisms exist for recruitment of one or the other of these X-family polymerases to sites of specific DNA lesions. To better understand the interrelationship between these enzymes in mammalian cells and their effect on important cellular phenotypes such as oxidative stress-induced mutagenesis, the availability of mouse fibroblasts cell lines with altered expression of these two polymerases could be invaluable.

Here, we examined the ability of two X-family polymerases, pol λ and pol β, to substitute for one another by isolating mouse embryonic fibroblast (MEF) cell lines with targeted deletions in each one or both polymerases. To avoid any confusion regarding a potential effect of DNA polymerase iota (pol ι), the cells were examined to ensure the wild-type form of the pol ι gene was present in the genome of each cell line. By using a neutral red viability assay and extracts prepared from these double knockout cell lines in combination with an *in vitro* BER assay, we revealed an increase in cellular hypersensitivity to DNA damaging agents and a decrease in BER capacity when compared to extract from cells containing a targeted deletion in one of the polymerases. These results, therefore, provided much-needed information documenting the backup role of pol λ in mammalian cell BER. Further, we found that both pol β and pol λ can interact with relevant DNA glycosylases, 8-oxoguanine-DNA glycosylase 1 (OGG1) and alkyadenine-DNA glycosylase (AAG). These interactions could be important in recruiting polymerases to sites of BER and aid in efficient step-to-step coordination.

## Results and Discussion

### Generation of the pol λ −/− and pol β −/− double knockout mouse embryonic fibroblast cell line

**Figure 1:**
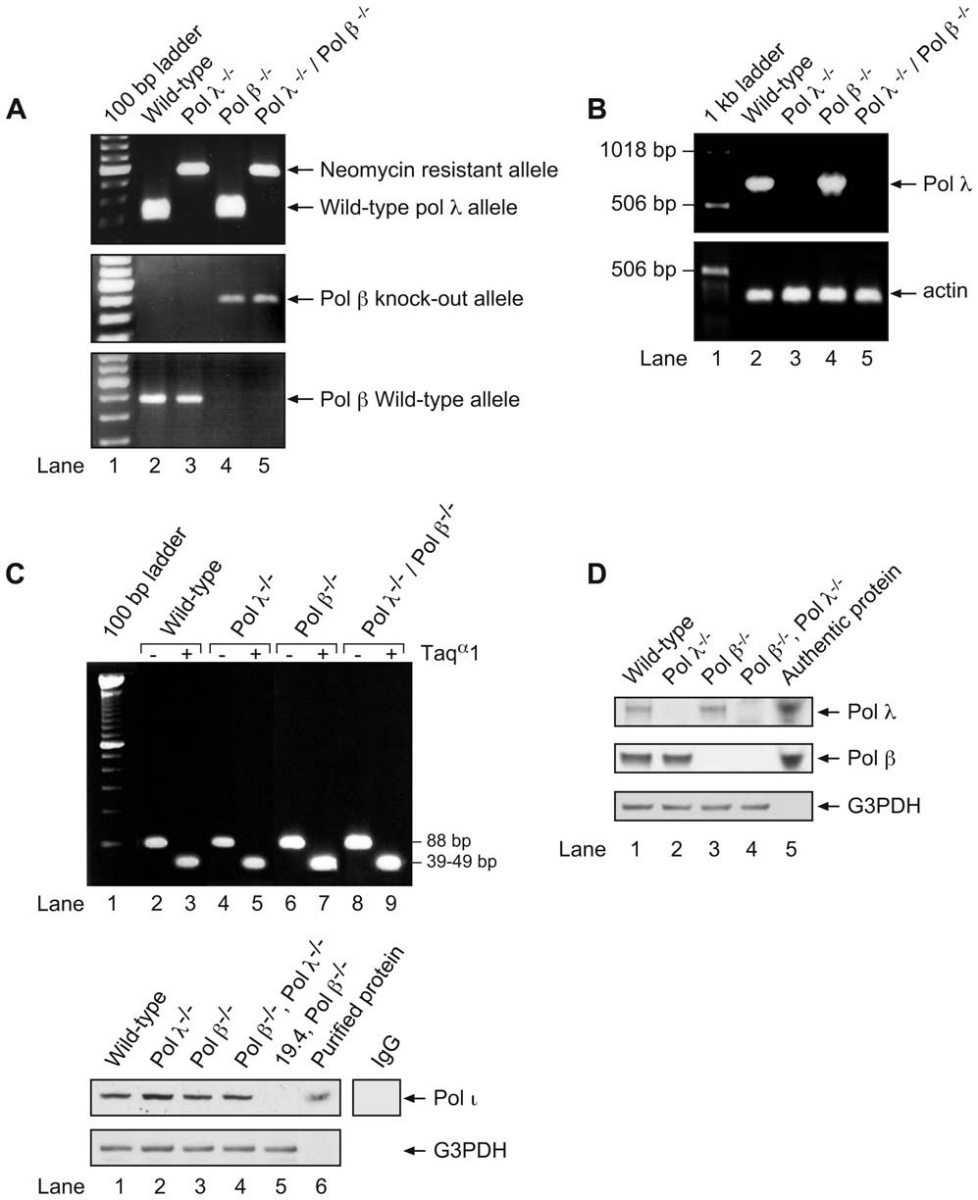
Verification of mouse cells with targeted disruption of pol λ and pol β genes and verification of pol ι expression. (*A*) Photographs of ethidium bromide stained agarose gels showing PCR-based analysis of genomic DNA. Targeted disruption of the pol λ gene (*top panel*) or pol β gene (*middle* and *bottom panels*) was studied in wild-type (*lane 2*), pol λ −/− (*lane 3*), pol β −/− (*lane 4*) and pol λ ^−/−^/pol β ^−/−^ double knockout (*lane 5*) MEF cells. For pol λ (*top panel*), the wild-type gene generates a 250 bp fragment and the pol λ knockout generates a 500 bp fragment, as indicated. For pol β, the pol β gene knockout generates a 520 bp fragment, and no fragment is generated for the wild-type alleles (*middle panel*). For pol β wild-type (*bottom panel*), a 443 bp fragment is generated, and no fragment is produced for the knockout allele. *Lane 1* shows migration of markers consisting of a 100 bp DNA ladder. (*B*) Analysis of cDNA for mRNA expression of pol λ. Photographs of ethidium bromide stained agarose gels showing amplification of cDNA. cDNA prepared from wild-type (*lane 2*), pol λ −/− (*lane 3*), pol β −/− (*lane 4*) and pol λ ^−/−^/pol β ^−/−^ double knockout (*lane 5*) MEF cells was amplified by specific primers targeted for pol λ (*top panel*) or actin (*bottom panel*). Sizes of the expected amplification products are indicated. *Lane 1*, migration of a marker DNA ladder. (*C*) Photograph of ethidium bromide stained agarose gels showing PCR-based analysis of the pol ι gene in genomic DNA. Genomic DNA prepared from wild-type (*lanes 2* and *3*), pol λ −/− (*lanes 4* and *5*), pol β −/− (*lanes 6* and *7*) and pol λ ^−/−^/pol β ^−/−^ double knockout (*lanes 8* and *9*) MEF cells was amplified using specific primers targeted for the pol ι gene, and an aliquot of the completed reaction mixture was digested with Taq^α^1. Sizes corresponding to the expected digestion products of the wild-type gene were observed in all four cell types (*lanes 3*, *5*, *7*, and *9*). *Lane 1* shows the migration of a DNA marker ladder. Two lower panels: Photographs of enhanced chemiluminescence (ECL) – stained immunoblots showing expression of pol ι (top panel) and G3PDH as a loading control (*bottom panel*). *Top panel*, immunoblotting with antibody to pol ι and wild-type (*lane 1*), pol λ −/− (*lane 2*), pol β −/− (*lane 3*), pol λ ^−/−^/pol β ^−/−^ (*lane 4*), and the 19.4 cell line- (pol ι minus (pol β −/−)) (*lane 5*) MEF cell extracts. *Lane 6* contained purified pol ι used as a marker and positive control. The lane shown under IgG corresponded to a non-immune IgG negative control with the pol λ ^−/−^/pol β ^−/−^ cell extract (i.e., blotting with non-immune IgG instead of anti-pol ι antibody); the pol ι region of the gel is shown. (D) Immunoblotting analysis for protein expression in wild-type (*lane 1*), pol λ −/− (*lane 2*), pol β −/− (*lane 3*) and pol λ ^−/−^/pol β ^−/−^ (*lane 4*) MEF cell extracts. Photographs of enhanced chemiluminescence-stained immunoblots showing expression of pol λ (*top panel*) or pol β (*2nd panel*) and G3PDH, as a loading control, (*bottom panel*). *Lane 5* contains the respective purified enzyme used as a marker and positive control. Experiments were conducted as described under Materials and Methods.

To further characterize the contributions of pol λ and pol β in mammalian BER, we bred pol λ^ +/−^/pol β ^+/−^ mice together. No animals carrying the pol β −/− genotype resulted in viable mice. Nevertheless, viable embryos could be isolated between embryonic day 14.5 to 16.5, and the various embryos were designated as: 1) wild-type for the two DNA polymerase genes; 2) pol λ −/−; 3) pol β −/−; and 4) pol λ ^−/−^/pol β ^−/−^ double knockout. We then prepared primary MEFs as described under Materials and Methods. PCR and RT-PCR were used to verify the DNA polymerase status of each cell type (Fig. 1). To assess the pol λ gene status by PCR of genomic DNA, sequence-specific primers were used to amplify the polymerase domain. Using these primers, a 250 bp PCR fragment was observed in cell lines that were wild-type for pol λ, while a 500 bp fragment resulted from the deletion and neomycin resistant allele (Fig. 1*A*, *top panel*). Similarly, to assess the pol β gene, separate PCR reactions were performed to detect the wild-type and knockout alleles. A 520 bp PCR fragment was amplified for the knockout allele (Fig. 1*A*, *middle panel*) and a 443 bp fragment was amplified for the wild-type allele (Fig. 1*A*, *bottom panel*). In assessing mRNA expression by RT-PCR (Fig. 1*B*), the pol λ transcript was observed in the wild-type and pol β −/− cells (*lanes 2* and *4*, *top panel*), but not in the two pol λ −/− cell types (Fig. 1*B*). These primary cells were SV40 T-antigen transformed and single-cell cloned. Immunoblotting analysis (Fig. 1*D*) confirmed that these cells with the pol λ −/− and pol λ ^−/−^/pol β^−/−^ double knockout genotypes were, indeed, negative for pol λ expression (*lanes 2* and *4*, *top panel*), whereas the wild-type and pol β −/− cells were positive (*lanes 1* and *3*, *top panel*). Similarly, cells with the pol β −/− genotype were negative for expression of pol β (*lanes 3* and *4*, *middle panel*), and the cells with pol β +/+ genotype were positive (*lanes 1* and *2*, *middle panel*). Finally, to avoid any confusion stemming from loss of pol ι, as was the case earlier with mice containing the strain 129 background [8], we assessed whether these current cell lines contained a wild-type copy of the pol ι gene. All four cell types were found to be wild-type for the pol ι gene, as observed by the ability of Taq^α^1 to digest the PCR fragment containing the wild-type sequence encoding a serine at position 27 (Fig. 1*C*). The expression of pol ι was further confirmed by immunoblotting of the extracts using antibody to pol ι (Fig. 1*C*). Extracts were analyzed from wild type, pol λ−/−, pol β −/−, and pol λ ^−/−^/pol β^−/−^ cells, along with a pol ι negative control; this control was the well-characterized pol β −/− cell line that is known to be deficient in pol ι, i.e., 19.4 [9], [10]. The results verified a similar level of expression of pol ι in all of the new cell lines prepared in this study.

### Comparison of extract-mediated BER for wild-type and knockout mouse cell lines

**Figure 2:**
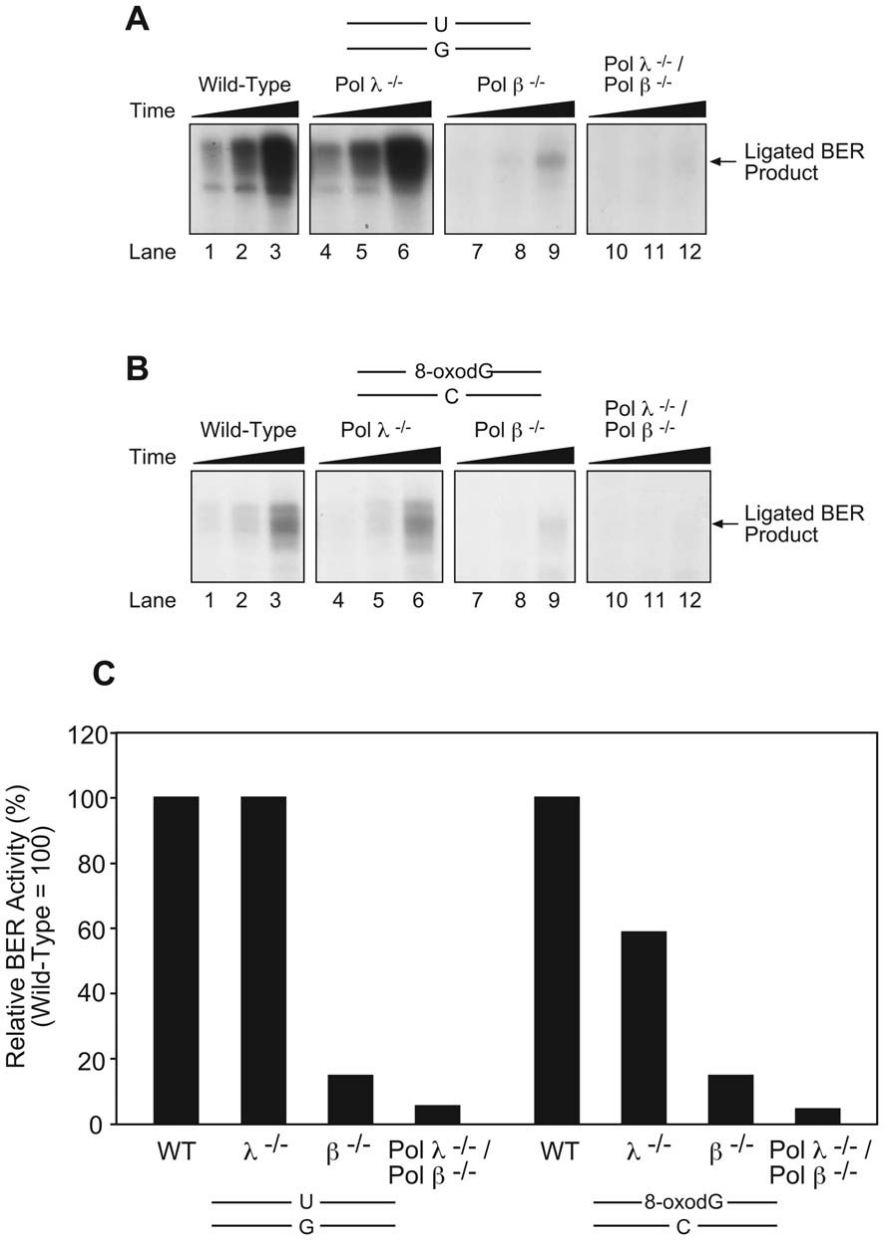
Pol λ ^−/−^/pol β ^−/−^ double knock-out cell extracts show strongly reduced *in vitro* BER activity. Experiments were conducted as described under Materials and Methods. *In vitro* BER time course experiments showing the capacity of wild-type (*lanes 1–3*), pol λ −/− (*lanes 4–6*), pol β −/− (*lanes 7–9*) and pol λ ^−/−^/pol β ^−/−^ (*lanes 10–12*) MEF cell extracts to repair a solitary base lesion in double-stranded DNA, the uracil (U) lesion opposite G (*A*) or the 8-oxoguanine (8-oxodG lesion opposite C (*B*). The schematic at the top in each panel illustrates the DNA substrate, and the reaction mixtures contained [α-^32^P]dCTP and [α-^32^P]dGTP, respectively. The BER reaction mixtures were incubated for 5 (*lanes 1*, *4*, *7 and 10*), 10 (*lanes 2*, *5*, *8*, and *11*) or 30 (*lanes 3*, *6*, *9* and *12*) min, respectively. Photographs of autoradiograms after denaturing PAGE are shown illustrating incorporation of [^32^P]dCMP (*A*) or [^32^P]dGMP (*B*) into the fully repaired DNA. The positions of the fully repaired and ligated BER products are indicated. (*C*) Quantification of the *in vitro* BER activity of the cell extracts used in panels *A* and *B*. Average values are shown for the respective extracts.

To understand the effect of the combined deletion of pol λ and pol β, we first examined the BER capacity of cell extracts. In experiments shown in Fig. 2, extract-mediated BER of model BER substrates, uracil-DNA and 8-oxodG-DNA, was studied. For both substrates, the extract from pol λ −/− cells was able to support BER of the lesion (Fig. 2*A*, *lanes 1–3* and *4–6*; Fig. 2*B*, lanes *1–3* and *4–6*); in contrast, the extract from pol β −/− cells was strongly deficient. This was consistent with earlier observations with pol λ −/− knockdown cells [4]. Interestingly, the extract from the double knockout cells was essentially devoid of *in vitro* BER, indicating a modest back up role of another polymerase in the absence of pol β (Fig. 2*A*, *lanes 10–12*; Fig. 2*B*, *lanes 10–12*). In the case of *in vitro* BER of the 8-oxodG lesion (Fig. 2*B*), extract from pol λ −/− cells was less active than extract from wild-type cells. Pol β −/− cells, on the other hand, retained very weak *in vitro* BER activity for the 8-oxodG lesion, and as noted the extract from the double knockout cells was essentially devoid of 8-oxodG *in vitro* BER activity. These results are consistent with the idea that pol β contributed the main DNA polymerase function in these extract-based BER reactions; nevertheless, in the absence of pol β pol λ, or another polymerase, was able to contribute modest activity. This modest activity disappeared in the double knockout extract, suggesting that it was due to pol λ.

### Sensitivity of mouse cell lines to DNA damaging agents

**Figure 3:**
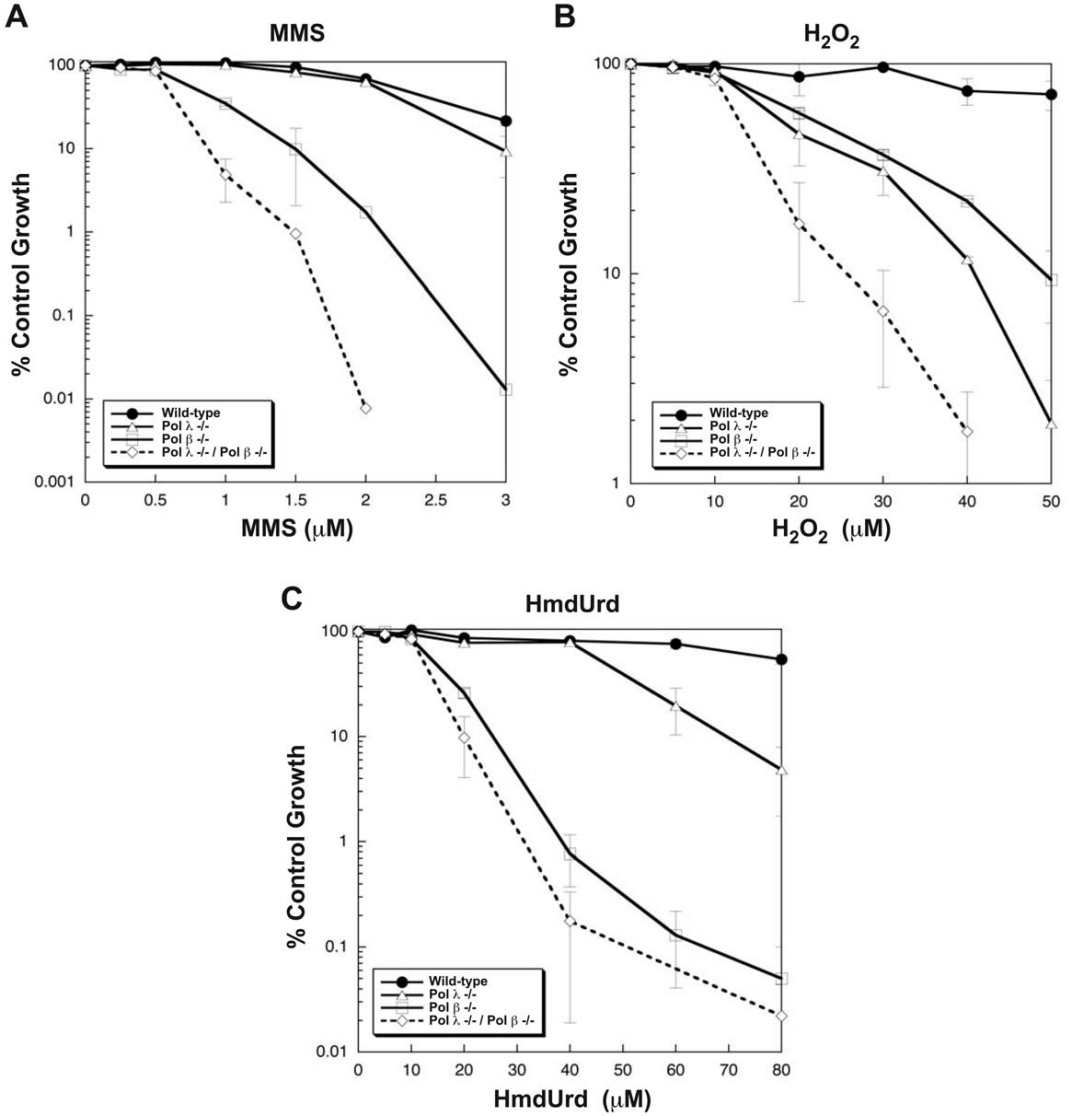
Sensitivity of single and double knockout MEF cell lines to DNA damaging agents. Experiments were conducted as described under Materials and Methods. Wild-type (*closed circles*) and pol λ −/− (*open triangles*), pol β −/− (*open rectangles*) and pol λ ^−/−^/pol β^−/−^ (*open diamonds*) cells were exposed to increasing concentrations of MMS for 1 h (*A*), H_2_O_2_ for 1 h (*B*) or HmdUrd for 24 h (*C*). Percent control growth was plotted for each data point, representing the mean values of triplicate experiments.

The double knockout cell line was examined for sensitivity to three DNA-damaging agents and compared in the same experiments with the wild-type and single knockout cell lines (Fig. 3). For treatment with methyl methanesulfonate, hydrogen peroxide and HmdUrd, the double knockout cells were more sensitive than the other three cell types. Since each of these agents produce DNA damage that is repaired by BER, these results thus indicate that the two X-family DNA polymerases are involved in BER mediated repair. When examining MMS sensitivity, the current mouse pol λ −/− cell line was not substantially more sensitive to MMS than the wild-type cell line except at the highest dose tested, whereas the pol β −/− cell line was hypersensitive to MMS treatment. This finding is consistent with previous studies in chicken DT40 cells [5]. Additionally, cells deficient in both pol λ and pol β were even more sensitive than cells deficient in pol β alone, which suggests that these enzymes behave synergistically and participate in repair of a common set of DNA lesions.

In the case of H_2_O_2_ toxicity, a different sensitivity profile was observed for the single and double knockout cells. Double knockout cells exhibited an additive hypersensitivity to H_2_O_2_ when compared to the single mutants, suggesting that pol λ and pol β mediated repair pathways act upon different sets of H_2_O_2_-induced DNA lesions. Again, this finding is consistent with previous studies in chicken DT40 cells [5]. The hypersensitivity of the pol β −/− cell line to H_2_O_2_ was similar to that found by others [11] and to that of late passage pol β −/− MEFs [1].

In contrast to earlier results with a different pol λ −/− cell line [4], the current pol λ −/− cell line was only modestly hypersensitive to HmdUrd, whereas the double knockout line was slightly, but significantly, more hypersensitive than the pol β −/− line (Fig. 3*C*). Overall, these results indicate that pol λ had a protective effect against the agents tested that was especially evident in the pol β-deficient background.

### Pols λ and β interact with the DNA glycosylases AAG and OGG1

Step-to-step coordination between the lesion removal step in BER and the subsequent DNA polymerase steps is an important, but poorly understood, feature of the BER pathway. Direct interaction between the BER DNA polymerases and DNA glycosylases could potentially enhance step-to-step coordination. We found earlier that pol λ was able to co-immunoprecipitate with the oxidized uracil DNA glycosylase termed SMUG 1 [4], and other studies had revealed interactions between pol β and the Neil DNA glycosylases [12]. In light of the results of the present study, we were curious to examine the possibility of co-immunoprecipitation of pol λ and pol β with the alkyladenine-DNA glycosylase and 8-oxoG-DNA glycosylases, i.e., the enzymes termed AAG and OGG1, respectively. The potential interaction of these two X-family DNA polymerases with these two upstream DNA glycosylases in the BER pathway could have important implications in the BER process.

**Figure 4:**
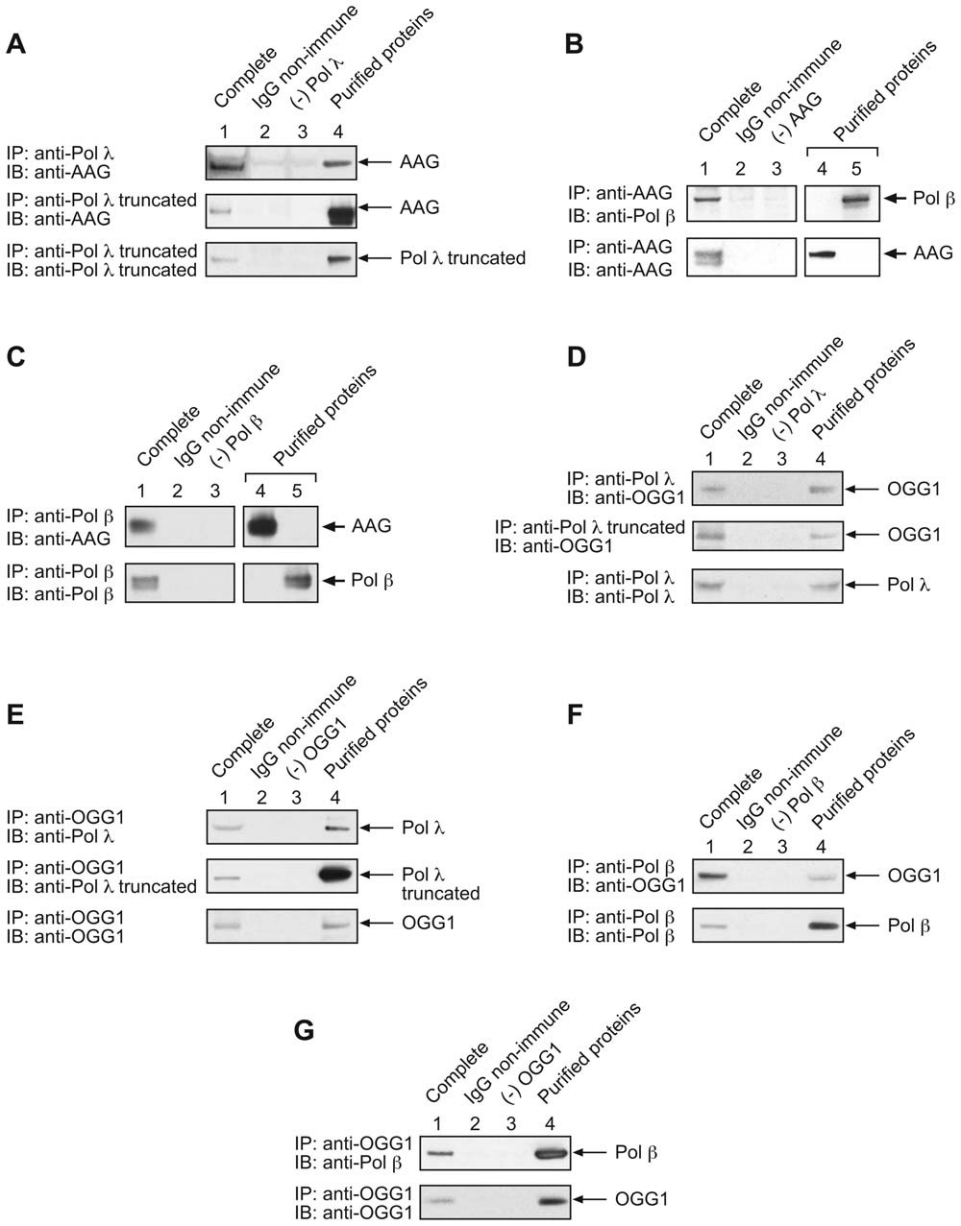
Purified AAG and OGG1 co-immunoprecipitate with both purified pol λ and pol β. Photographs of enhanced chemiluminescence-stained immunoblots are shown. (*A*) *Top panel*: immunoprecipitation from a mixture of 1.5 µM each of full-length pol λ and AAG using anti-pol λ antibody (*lane 1*); control with AAG and full-length pol λ using non-immune IgG (*lane 2*); control with AAG and anti-pol λ antibody (*lane 3*). In *lane 4*, AAG was added as a positive control for immunoblotting. In the *bottom* and *middle panels* of *A*, full-length pol λ was substituted with a truncated form of pol λ containing the polymerase domain only: Immunoblotting to detect AAG (*top and middle panels*); immunoblotting to detect the truncated form of pol λ (*bottom panel*). (*B*) Immunoprecipitation with a mixture of 1.5 µM each pol β and AAG using anti-AAG antibody (*lane 1*); control with AAG, pol β and non-immune IgG (*lane 2*); control with pol λ and anti-AAG antibody (*lane 3*). In *lanes 4* and *5*, AAG and pol β, respectively, were added as a positive control for immunoblotting. Immunoblotting to detect pol β (*top panel*); immunoblotting to detect AAG (*bottom panel*). (*C*) Immunoprecipitation with a mixture of 1.5 µM each pol β and AAG and polyclonal anti-pol β antibody (*lane 1*); control with AAG, pol β and non-immune IgG (*lane 2*); and control with AAG and anti-pol β antibody (*lane 3*). In *lanes 4* and *5*, AAG and pol β, respectively, were added as immunoblotting positive controls: Immunoblotting to detect AAG (*top panel*); immunoblotting to detect pol β (*bottom panel*). (*D*) Top and bottom panels: Immunoprecipitation with a mixture of 1.5 µM each full-length pol λ and OGG1 and polyclonal anti-pol λ antibody (*lane 1*); control with OGG1 and full-length pol λ and non-immune IgG (*lane 2*); and control with OGG1 and anti-pol λ antibody (*lane 3*). In *lane 4*, OGG1 or pol λ was added as an immunoblotting positive control. In the middle panel, full-length pol λ was substituted with a truncated form of pol λ containing the polymerase domain: Immunoblotting to detect OGG1 (*top and middle panels*); immunoblotting to detect pol λ (*bottom panel*). (*E*) Top and bottom panels: Immunoprecipitation with a mixture of 1.5 µM each purified full-length pol λ and OGG1 and polyclonal anti-OGG1 antibody (*lane 1*); control with OGG1 and full-length pol λ and non-immune IgG (*lane 2*); and control with pol λ and anti-OGG1 antibody (*lane 3*). In *lane 4*, pol λ or OGG1 was added as an immunoblotting positive control. In the middle panel, full-length pol λ was substituted with a truncated form of pol λ containing the polymerase domain: Immunoblotting to detect pol λ (*top panel*); immunoblotting to detect the truncated form of pol λ (*middle panel*); immunoblotting to detect OGG1 (*bottom panel*). (*F*) Immunoprecipitation with a mixture of 1.5 µM each pol β and OGG1 and polyclonal anti-pol β antibody (*lane 1*); control with OGG1 and pol β and non-immune IgG (*lane 2*); and control with OGG1 and anti-pol β antibody (*lane 3*). In *lane 4*, purified OGG1 or pol β was added as an immunoblotting positive control: Immunoblotting to detect OGG1 (*top panel*); immunoblotting to detect pol β (*bottom panel*). (*G*) Immunoprecipitation with a mixture of 1.5 µM each pol β and OGG1 and polyclonal anti-OGG1 antibody (*lane 1*); control with OGG1 and pol β and non-immune IgG (*lane 2*), and control with purified pol β and anti-OGG1 antibody (*lane 3*). In *lane 4*, purified OGG1 or pol β was added as an immunoblotting positive control: Immunoblotting to detect pol β (*top panel*); immunoblotting to detect OGG1 (*bottom panel*).

Two forms of pol λ were used, the full-length enzyme and a truncated version corresponding to the polymerase domain but lacking the BRCT domain. First, we found that the pol λ antibody was able to co-immunoprecipitate AAG from a mixture of purified AAG and either full-length pol λ or the truncated form of pol λ (Fig. 4*A*). The non-immune IgG and minus AAG controls were negative in these experiments. Reciprocal immunoprecipitations with anti-AAG antibody were not successful.

Similarly, the pol β antibody was able to co-immunoprecipitate AAG, and in reciprocal experiments with antibody against AAG, pol β was co-immunoprecipitated (Fig. 4*C* and *B*, respectively). Next, we found that the pol λ antibody was able to co-immunoprecipitate OGG1 from a mixture of purified OGG1 and either full-length or truncated pol λ (Fig. 4*D*). When the experiments were conducted with antibody to OGG1, pol λ and the truncated form of pol λ were co-immunoprecipitated (Fig. 4*E*). The negative controls using non-immune IgG did not result in precipitation of polymerase of glycosylase, as expected (Figs. 4*D* and *E*, *lanes 2* and *3*). Since the truncated form of pol λ was able to co-immunoprecipitate AAG and OGG1, the BRCT domain was not required for these interactions. Similar experiments with antibody to pol β revealed co-immunoprecipitation of OGG1 from a mixture of purified pol β and OGG1 (Fig. 4*F*). Reciprocal experiments with antibody to OGG1 revealed co-immunoprecipitation of pol β (Fig. 4*G*). The negative controls failed to show co-immunoprecipitation in each case, as expected (Figs. 4*F* and *G*, *lanes 2* and *3*).

**Figure 5:**
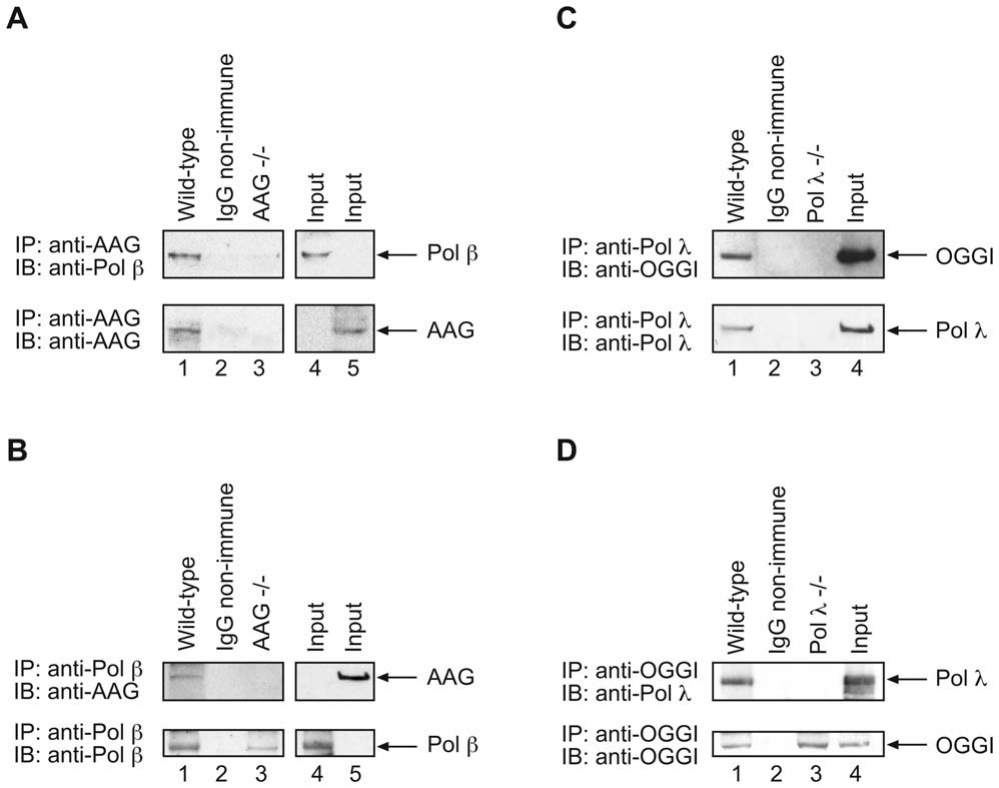
Extract-based co-immunoprecipitations. Experiments were conducted as described under Materials and Methods. Photographs of ECL-stained immunoblots are shown. *A*, Immunoprecipitations (IP) of MEF cell extracts with anti-AAG antibody or control non-immune IgG: *Top panel*, immunoblotting to detect pol β; *bottom panel*, immunoblotting to detect AAG. In the *top panel*, IP incubations were performed using matched wild-type (AAG +/+) (*lanes 1 and 2*) and AAG −/− cell extracts (*lane 3*) and anti-AAG or non-immune IgG (*lane 2*); immonoblotting was with anti-pol β antibody. In the *bottom panel*, immunoblotting was with anti-AAG antibody. In *lane 4*, 50 µg of AAG +/+ extract (1/20^th^ of the IP input) was subject to SDS-PAGE as a marker for AAG. *B*, Immunoprecipitations of MEF cell extracts as in *A* with anti-pol β antibody or non-immune IgG. *Top panel*, immunoblotting to detect AAG; *bottom panel*, immunoblotting to detect pol β. *Panels C* and *D* illustrate pol λ and OGG1 co-immunoprecipitation from MEF cell extracts, as in *panels A* and *B*. IP incubations were performed with matched pol λ+/+ cell extract (*lanes 1 and 2*) and pol λ−/− cell extract (*lane 3*), as shown in the figure. Similarly, non-immune IgG was used as a negative control (*lanes 2*). *C. Top panel*, immunoblotting to detect OGG1; *bottom panel*, immunoblotting to detect pol λ. *D*. *Top panel*, immunoblotting to detect pol λ; *bottom panel*, immunoblotting to detect OGG1. In *lane 4*, 50 µg of pol λ+/+ extract was subject to SDS-PAGE as a marker.

In addition to these results with purified enzymes, we examined extracts from wild-type (AAG +/+) and AAG −/− mouse fibroblasts cell lines to evaluate co-immunoprecipitations by the antibodies to AAG and pol β (Fig. 5*A* and *B*, respectively). Antibody to AAG was able to co-immunoprecipitate pol β and AAG, and in reciprocal experiments with antibody against pol β, AAG was co-immunoprecipitated. Immunoprecipitations were not observed with extract from AAG −/− cells or with non-immune IgG, as expected (Fig. 5*A* and *B*, *lane 3*). Next, cell extracts were prepared from the wild-type (pol λ +/+) and pol λ −/− mouse fibroblasts cell lines to evaluate co-immunoprecipitations of pol λ and OGG1 (Fig. 5*C* and *D*, respectively). Antibody to pol λ co-immunoprecipitated OGG1 and pol λ. In reciprocal experiments with antibody to OGG1, pol λ was co-immunoprecipitated along with OGG1. In each case, the negative control with IgG non-immune antibodies, no polymerases or glycosylases immunoprecipitated, as expected. These data are consistent with the results obtained with the purified enzymes suggesting interactions between these two X family DNA polymerases and the respective DNA glycosylases for removal of alkylation base damage and oxidative base damage, 8-oxodG.

### Conclusions

Our results with pol β and pol λ double knockout cells indicated that these two X-family polymerases have roles in protection of MEF cells against exposure to three different genotoxic agents: MMS, H_2_O_2_ and HmdUrd. The additive effect for protection against H_2_O_2_, as opposed to overlapping roles, was surprising, since pol β and pol λ share many similarities in their *in vitro* BER-related activities or biochemical properties. Yet, the enzymes are products of different genes and have different primary structures. Obviously, differences, including the pol λ BRCT domain that is not found in pol β, in cellular compartmentalization, in partner protein interactions or nuclear expression levels for the two polymerases may account for the distinct cellular phenotypes of the cell lines with pol β or pol λ deficiencies. These ideas are consistent with recent observations by Maga et al. regarding the effects of differential expression of these two polymerases and of differential 8-oxodG lesion bypass efficiency in the presence of auxiliary factors [13].

Our results also indicated that both of these X-family DNA polymerases can directly interact with the respective DNA glycosylases that could help direct the polymerases to the vicinity of alkylation-induced and oxidation-induced base damage, respectively. These types of DNA damage are thought to be repaired by BER *in vivo*, and the cellular hypersensitivity results reported here for the double knockout cells are consistent with this interpretation. It should be interesting in the future to examine the biological consequence of disrupting the respective polymerase-glycosylase interactions upon exposures leading to alkylation-induced and oxidation-induced base lesions.

## Materials and Methods

### Cell lines

Pol λ −/− [14] and pol β +/− [15] mice were previously generated from embryonic stem cells after targeted gene knockout in mice containing a C57BL/6 background. Mice were maintained at the National Institute of Environmental Health Sciences' animal facility in specific pathogen-free conditions and the Animal Care and Use committee approved all animal protocols. Initially, pol λ −/− and pol β +/− mice were crossed to produce pol λ ^+/−^/pol β ^+/−^ mice. These heterozygous mice were then bred and embryos were collected corresponding to the following genotypes: Wild-type (pol λ +/+ and pol β +/+), pol λ −/− (pol λ −/− and pol β +/+), pol β −/− (pol λ +/+ and pol β −/−), and pol λ ^−/−^/pol β ^−/−^. Primary mouse embryonic fibroblasts (MEFs) were prepared from isolated embryos as previously described [16].

### Purified enzymes and antibodies

Recombinant full-length human DNA polymerases β and λ were purified as described by Beard and Wilson [17] and Braithwaite et al. [4], respectively. The truncated form of pol λ, lacking the BRCT domain, was a gift from Miguel García-Díaz. The purified human DNA glycosylase alkyladenine-DNA glycosylase (AAG) was prepared as described [18]. The mouse 7,8-dihydro-8-oxoguanine (8-oxodG)-DNA glycosylase 1 (OGG1) was obtained from Arthur Grollman [19]. Antibody to monoclonal (18 S) and polyclonal antibodies against pol β were developed as described previously [20], whereas antibodies specific for full-length human pol λ were raised by immunization of rabbits as described previously [4], [21]. Truncated pol λ antibody was from Miguel García-Díaz and Luis Blanco (Centro de Biología Molecular Severo Ochoa). G3PDH monoclonal antibody was from Alpha Diagnostic Intl. Inc. (San Antonio, TX). Goat polyclonal anti-DNA polymerase *ι* antibody was from Santa Cruz (Santa Cruz, CA). Mouse and rabbit IgG secondary antibodies were goat anti-mouse IgG horseradish peroxidase conjugate and goat anti-rabbit IgG horseradish peroxidase conjugate, respectively (Bio-Rad, Hercules, CA).

### Transformation of primary mouse embryonic fibroblast cells

Sub-confluent cultures of primary MEFs were transfected with the pSVAgt plasmid, which expresses SV40 wild-type viral T-antigen (generously provided by Robert Sobol). Primary MEFs were incubated in Lipofectamine solution (Invitrogen) containing pSVAgt plasmid DNA for 17 h at 37°C. The Lipofectamine solution was then replaced with growth medium, and the cells were cultured at 37°C for an additional 48 h. After reaching confluency, cells were repeatedly split at a ratio of 1∶20 until the characteristics of transformed cells were achieved (e.g., fast growing, change in cell morphology, lack of contact inhibition). Subsequently, cells were single-cell cloned by plating at a density of 0.1 – 1 cell per well in a 96-well dish. Four clones were selected from each cell line.

### Cell extract preparation

Cell extracts were prepared as previously described [22]. Briefly, cells were washed twice with PBS at room temperature, detached by scraping, pelleted and resuspended in Buffer I (10 mM Tris-Cl, pH 7.8, 200 mM KCl and protease inhibitors). Subsequently, an equal volume of Buffer II (10 mM Tris-Cl, pH 7.8, 200 mM KCl, 2 mM EDTA, 40% glycerol, 0.2% Nonidet P-40 and 2 mM dithiothreitol) was added. The suspension was rotated at 4°C for 1 h, and the resulting extracts were centrifuged (20,800×g) for 10 min at 4°C. Supernatant fractions were recovered for use in *in vitro* BER assays and immunoblotting experiments. The protein concentrations of extracts were determined by the Bio-Rad protein assay using bovine serum albumin as standard.

### Co-immunoprecipitations using cell extracts

Mouse fibroblasts cell lines of various genotypes as follows, wild-type or pol λ+/+ and pol λ−/−, and wild-type or AAG +/+ and −/−, were washed and harvested as described above. Cell pellets were resuspended in lysis buffer (50 mM Tris-HCl, pH 7.5, 150 mM NaCl, 25 mM NaF, 0.1 mM sodium orthovanadate, 0.2% (v/v) Triton X-100, and 0.3% (v/v) Nonidet P-40) containing protease inhibitors (0.1 mM phenylmethylsulfonyl fluoride, 1 mg/ml aprotinin, and 5 mg/ml leupeptin) and then processed as described previously [4]. Briefly, the cell suspensions were incubated on ice for 30 min and centrifuged at 20,800×*g* for 30 min at 4°C, and the supernatant fractions (equal amount of total protein) were used for the co-immunoprecipitation assays. The protein concentrations of extracts were determined by the Bio-Rad protein assay using bovine serum albumin as standard.

### Genotyping by PCR

Genomic characterization of wild-type, pol λ-deficient, pol β-deficient, and wild-type pol ι embryos and cell lines were performed by polymerase chain reactions (PCR) as previously described [8], [14], [15]. For pol β, 5′- AAGGACGGAAGGTGGAGGGAGAGCTAATGC-3′ (MBFOR1) and 5′- CTGGCTCACGTTCTTCTCAAAGTTTGCGAG-3′ (MBEX2) primers were used for detection of the pol β null allele, and 5′- ATAACAATTTATGCCCAAACGAAT-3′ (MBpolWTA) and 5′-TCTGATTTAGAGCCCGAGATG-3′ (MBpolWTB) for detection of the wild-type allele. Genotyping of mice, embryos and cell lines for pol λ was performed with the following primers: 5′-GCTCCATATGGTTGCTGGGC-3′ (pol λ upstream primer), 5′- CAGCTCCCCAGATGTTGGAG-3′ (wild-type primer) and 5′- CATAGCGTTGGCTACCCGTG-3′ (neoR primer; Integrated DNA Technologies). To identify wild-type and mutant alleles for pol ι, 50 ng of genomic DNA isolated from embryos or cell lines was amplified using primers: 5′-CAGTTTGCAGTCAAGGGCC (forward) and 5′-TCGACCTGGGCATAAAAGC (reverse) in a 50 µl reaction volume. Following PCR, a 25 µl aliquot of the completed reaction was removed and treated with Taq^α^I at 65°C for 1 h. After incubation, the treated and untreated portions of the PCR reactions were separated by 2% agarose gel electrophoresis. Cell lines containing the wild-type pol ι sequence are digested by Taq^α^I to yield 39 and 49 base pair fragments. Genes containing the mutant sequence with a C→A substitution at the serine 27 codon do not contain the restriction enzyme site and, therefore are not cut by the enzyme.

### RT-PCR of cDNA

Total RNA was isolated from cells using TRIZOL Reagent (Invitrogen), and cDNA was synthesized from total RNA using SuperScript first-strand synthesis system for RT-PCR (Invitrogen) per the manufacturer's instructions. For PCR reactions, cDNA templates were subjected to PCR with Taq polymerase using gene specific primers (for pol λ: GCC CAG CTC AGC TCA GAG GAT GAA and CGT CGG TAA GAG CCA CAA GCC ACA; for actin: β-actin mRNA primers from Ambion).

### Immunoblotting

After suspending 50 µg of cell extract in SDS sample buffer, proteins were heated at 95°C for 5 min and separated by 4–12% SDS-polyacrylamide gel electrophoresis. The proteins were then transferred to nitrocellulose membranes, and these were blocked with 5% nonfat milk in Tris-buffered saline containing 0.05% (v/v) Tween 20 (TBST). After incubation with primary antibodies against DNA polymerases λ or β, or ί the membranes were washed with TBST and secondary antibodies were added. After three washes with TBST, enhanced chemiluminescence was used to detect the peroxidase conjugate by exposure to X-ray film.

### *In vitro* BER assay

The DNA substrates were 35-mer double-stranded oligonucleotides. They contained either a site-specific uracil (U) modification at position 15 in the sequence 5′-CTGCAGCTGATGCG*X*CGTACGGATCCCCGGGTAC-3′, where “*X*” denotes the position of the uracil, or a site-specific 8-oxoguanine modification at position 17 in the sequence 5′-CTGCAGCTGATGCGCC*X*TACGGATCCCCGGGTAC-3′, where “*X*” denotes the position of 8-oxoguanine (8-oxodG). These oligonucleotides were purchased from The Midland Certified Reagent Co. (Midland, TX). The lesion-containing oligonucleotides were annealed to a complementary strand that contained the sequence 5′-GTACCCGGGGATCCGTACGGCGCATCAGCTGCAG. Time courses for *in vitro* BER reactions were obtained by incubating the 35-base pair oligonucleotide duplex (250 nM) in a final volume of 10 µl with 10 µg of MEF cell extract at 37°C. The reaction mixtures also contained 25 mM Tris, pH 7, 60 mM NaCl, 2 mM dithiothreitol, 0.2 mM EDTA, 1 mg/ml bovine serum albumin, 10% (v/v) glycerol and ^32^P-labeled dNTP. At various times, 3 µl aliquots of the reaction mixture were removed for analysis, and BER products were observed as described previously [1], [4], [22]. Briefly, the gels were scanned by PhosphorImager, and the ligated BER products were quantified using ImageQuant software. Arbitrary units from the analysis were used to calculate relative BER activity, with the activity of wild-type extract taken as 100 percent.

### Neutral red viability cytotoxicity assays

Neutral red viability assays were performed using a FLUOstar OPTIMA plate reader (BMG LABTECH) as previously described [23], with minor modifications. After plating the cells at a density of 3,125 cells per well in 48-well dishes, cells were exposed to increasing concentrations of H_2_O_2_ for 1 h, methyl methanesulfonate (Sigma) for 1 h, or 5-hydroxymethyldeoxyuridine (HmdUrd) for 24 h. Cells were washed with Hanks' balanced salt solution and allowed to grow for 4–5 days in growth medium at 37°C in a 10% CO_2_ incubator. Once the cells were 80–90% confluent, they were incubated in medium containing 40 µg/ml neutral red for 3 h at 37°C in a 10% CO_2_ incubator. After the incubation, the cells were washed with Hanks' balanced salt solution and then with neutral red assay fixative (0.1% CaCl_2_ in 0.5% formaldehyde). Finally, the cells were exposed to neutral red assay solubilization solution (1% acetic acid in 50% ethanol). The mixture was incubated at room temperature for 15 min without agitation and then for an additional 30 min with agitation. Absorbance at 540 nm was measured using a FLUOstar OPTIMA plate reader (BMG LABTECH). Results from triplicate experiments for each concentration were counted, and the averages are expressed as the “% Control Growth” [(OD_540_ for treated cells)/(OD_540_ for control cells) * 100].

### Co-immunoprecipitation assays with purified proteins

Co-immunoprecipitations of purified DNA polymerases (pol λ and pol β) and DNA glycosylases (OGG1 and AAG) were performed in the presence of binding buffer (25 mM Tris-HCl, pH 8, 10% glycerol, 100 mM NaCl, 0.01% Nonidet P-40) containing protease inhibitors (0.1 mM phenylmethylsulfonyl flouride, 1 mg/ml aprotinin and 5 mg/ml leupeptin), as previously described [24]. A 1.5 µM equimolar mixture of DNA polymerase (pol λ, truncated pol λ or pol β) and DNA glycosylase (OGG1 or AAG) as specified, was combined with either anti-pol λ, anti-pol β, anti-OGG1 or anti-AAG antibody in a final volume of 50 µl. The mixture was incubated with rotation for 4 h at 4°C. Antibody-containing protein complexes then were adsorbed onto protein A-sepharose CL-4B beads (Amersham Biosciences) and protein G-agarose beads (Roche Molecular Diagnostics) (1∶1) or anti-rabbit IgG; Ip beads (eBioscience) were used when both immunoprecipitation and immunoblotting were performed with primary antibodies to rabbit, by incubation overnight at 4°C in a final volume of 500 µl of binding buffer. The beads were then washed with binding buffer containing protease inhibitors, suspended in SDS sample buffer, and heated for 5 min at 95°C. Soluble proteins were then recovered, separated by 4–12% SDS-PAGE and transferred onto nitrocellulose membranes. The membranes were blocked in 5% milk in TBST. For immunoblotting, the membranes were incubated with primary antibodies against pol λ, pol β, OGG1 or AAG, respectively. The membranes were then washed with TBST, submerged in binding buffer, and the secondary peroxidase-conjugated AffiniPure goat anti-rabbit IgG (H+L) antibody (Bio-Rad) or anti-rabbit IgG ‘True Blot’ HRP conjugated antibody (eBioscience) was added. After three washes with TBST, enhanced chemiluminescence (Perkin Elmer) was used to detect the bound peroxidase conjugate by exposure to X-ray film. To visualize co-precipitation of any interacting protein, the exposed nitrocellulose membrane was incubated in Pierce stripping buffer for 10 min at room temperature followed by 10 min at 37°C (to remove the primary antibody). The membrane was then blocked again in 5% milk in TBST, and finally it was immunoblotted as described above with the appropriate primary and secondary antibodies.

### Co-immunoprecipitation assays with mouse fibroblasts cell extracts

The co-immunoprecipitation incubations with matched wild-type or pol λ+/+ or pol λ−/− cell extracts were performed by mixing 1 mg of extract with 0.7 mg of rabbit non-immune IgG, anti-pol λ polyclonal antibody, or anti-OGG1 polyclonal antibody. The extract protein-antibody mixture was incubated at 4°C with rotation for 4 h, and immunocomplexes were then adsorbed onto TrueBlot anti-rabbit IgG IP beads (eBioscience, San Diego, CA) by incubating the mixture overnight at 4°C. Next, the beads were washed four times with lysis buffer containing protease inhibitors (0.1 mM phenylmethylsulfonyl fluoride, 1 mg/ml aprotinin, and 5 mg/ml leupeptin), resuspended in SDS sample buffer, and heated for 5 min. The soluble proteins were collected after centrifugation and separated by 4–12% NuPAGE Novex BisTris PAGE. After transfer to nitrocellulose membrane filter, proteins were detected as previously described using anti-OGG1 antibody (1∶1000 dilution) or anti-pol λ antibody (1∶5000) as a primary probe and goat anti-rabbit IgG TrueBlot conjugated to horseradish peroxidase (1∶5000 dilution) as the secondary antibody [4]. Immobilized horseradish peroxidase activity was detected by enhanced chemiluminescence. The nitrocellulose filter was then stripped by incubation in a buffer containing 6.25 mM Tris-HCl, pH 6.8, 100 mM β-mercaptoethanol and 1% (v/v) SDS for 30 min at 50°C followed by two washes with TBST at room temperature. The presence of pol λ was confirmed by incubating the membrane with rabbit anti-pol λ polyclonal antibody. Similarly, the cell extract was immunoprecipitated with anti-OGG1 polyclonal antibody to detect pol λ. After stripping the filter, the presence of OGG1 was confirmed using anti-OGG1 antibody. Co-immunoprecipitation incubations with matched wild-type (AAG +/+) and AAG −/− cell extracts were performed by mixing 1 mg of extract with 0.7 mg of rabbit non-immune IgG, anti-AAG polyclonal antibody or anti-pol β polyclonal antibody. The immunoprecipitation protocol as described above was followed and proteins were detected using anti-pol β antibody (1∶1000 dilution) or anti-AAG antibody (1∶1000) as primary probe and goat anti-rabbit IgG TrueBlot conjugated to horseradish peroxidase (1∶5000 dilution) as the secondary antibody. After stripping the filters, the presence of AAG was confirmed by incubating the membrane with rabbit anti-AAG polyclonal antibody. Similarly, the cell extract was immunoprecipitated with anti-pol β polyclonal antibody to detect AAG. After stripping the filter, the presence of pol β was confirmed using anti-pol β antibody.

## References

[1] Horton JK, Baker A, Berg BJ, Sobol RW, Wilson SH (2002). Involvement of DNA polymerase beta in protection against the cytotoxicity of oxidative DNA damage.. DNA Repair (Amst).

[2] Sobol RW, Kartalou M, Almeida KH, Joyce DF, Engelward BP (2003). Base excision repair intermediates induce p53-independent cytotoxic and genotoxic responses.. J Biol Chem.

[3] Sung JS, Demple B (2006). Roles of base excision repair subpathways in correcting oxidized abasic sites in DNA.. FEBS J.

[4] Braithwaite EK, Kedar PS, Lan L, Polosina YY, Asagoshi K (2005). DNA polymerase lambda protects mouse fibroblasts against oxidative DNA damage and is recruited to sites of DNA damage/repair.. J Biol Chem.

[5] Tano K, Nakamura J, Asagoshi K, Arakawa H, Sonoda E (2007). Interplay between DNA polymerases beta and lambda in repair of oxidation DNA damage in chicken DT40 cells.. DNA Repair (Amst).

[6] Crespan E, Hubscher U, Maga G (2007). Error-free bypass of 2-hydroxyadenine by human DNA polymerase lambda with Proliferating Cell Nuclear Antigen and Replication Protein A in different sequence contexts.. Nucleic Acids Res.

[7] Shimazaki N, Yazaki T, Kubota T, Sato A, Nakamura A (2005). DNA polymerase lambda directly binds to proliferating cell nuclear antigen through its confined C-terminal region.. Genes Cells.

[8] McDonald JP, Frank EG, Plosky BS, Rogozin IB, Masutani C (2003). 129-derived strains of mice are deficient in DNA polymerase iota and have normal immunoglobulin hypermutation.. J Exp Med.

[9] Sobol RW (2007). DNA polymerase beta null mouse embryonic fibroblasts harbor a homozygous null mutation in DNA polymerase iota.. DNA Repair (Amst).

[10] Poltoratsky V, Horton JK, Prasad R, Beard WA, Woodgate R (2008). Negligible impact of pol iota expression on the alkylation sensitivity of pol beta-deficient mouse fibroblast cells.. DNA Repair (Amst).

[11] Fortini P, Pascucci B, Belisario F, Dogliotti E (2000). DNA polymerase beta is required for efficient DNA strand break repair induced by methyl methanesulfonate but not by hydrogen peroxide.. Nucleic Acids Res.

[12] Wiederhold L, Leppard JB, Kedar PS, Karimi-Busheri F, Rasouli-Nia A (2004). AP endonuclease-independent DNA base excision repair in human cells.. Molecular Cell.

[13] Maga G, Villani G, Crespan E, Wimmer U, Ferrari E (2007). 8-oxo-guanine bypass by human DNA polymerases in the presence of auxiliary proteins.. Nature.

[14] Bertocci B, De Smet A, Flatter E, Dahan A, Bories JC (2002). Cutting edge: DNA polymerases mu and lambda are dispensable for Ig gene hypermutation.. J Immunol.

[15] Sobol RW, Horton JK, Kuhn R, Gu H, Singhal RK (1996). Requirement of mammalian DNA polymerase-beta in base-excision repair.. Nature.

[16] Robertson EJ, Rickwood D, Hames BD (1987). Embryo-derived stem cells.. Teratocarcinomas and Embryonic Stem Cells: A Practical Approach.

[17] Beard WA, Wilson SH (1995). Purification and domain-mapping of mammalian DNA polymerase beta.. Methods in Enzymology.

[18] O'Brien PJ, Ellenberger T (2003). Human alkyladenine DNA glycosylase uses acid-base catalysis for selective excision of damaged purines.. Biochemistry.

[19] Zharkov DO, Rosenquist TA, Gerchman SE, Grollman AP (2000). Substrate specificity and reaction mechanism of murine 8-oxoguanine-DNA glycosylase.. J Biol Chem.

[20] Srivastava DK, Rawson TY, Showalter SD, Wilson SH (1995). Phorbol ester abrogates up-regulation of DNA polymerase beta by DNA-alkylating agents in Chinese hamster ovary cells.. J Biol Chem.

[21] Harlow E, Lane D (1988). Antibodies: A Laboratory Manual..

[22] Braithwaite EK, Prasad R, Shock DD, Hou EW, Beard WA (2005). DNA polymerase lambda mediates a back-up base excision repair activity in extracts of mouse embryonic fibroblasts.. J Biol Chem.

[23] Repetto G, del Paso Z, Zurita JL (2008). Neutral red uptake assay for the estimation of cell viability/cytotoxicity.. Nature Protocols.

[24] Kedar PS, Kim SJ, Robertson A, Hou E, Prasad R (2002). Direct interaction between mammalian DNA polymerase beta and proliferating cell nuclear antigen.. J Biol Chem.

